# How ERAP1 and ERAP2 Shape the Peptidomes of Disease-Associated MHC-I Proteins

**DOI:** 10.3389/fimmu.2018.02463

**Published:** 2018-10-30

**Authors:** José A. López de Castro

**Affiliations:** Centro de Biología Molecular Severo Ochoa (CSIC-UAM), Madrid, Spain

**Keywords:** MHC, inflammatory diseases, ERAP, antigen processing, ankylosing spondylitis, uveitis, Behçet's disease, psoriasis

## Abstract

Four inflammatory diseases are strongly associated with Major Histocompatibility Complex class I (MHC-I) molecules: birdshot chorioretinopathy (HLA-A^*^29:02), ankylosing spondylitis (HLA-B^*^27), Behçet's disease (HLA-B^*^51), and psoriasis (HLA-C^*^06:02). The endoplasmic reticulum aminopeptidases (ERAP) 1 and 2 are also risk factors for these diseases. Since both enzymes are involved in the final processing steps of MHC-I ligands it is reasonable to assume that MHC-I-bound peptides play a significant pathogenetic role. This review will mainly focus on recent studies concerning the effects of ERAP1 and ERAP2 polymorphism and expression on shaping the peptidome of disease-associated MHC-I molecules in live cells. These studies will be discussed in the context of the distinct mechanisms and substrate preferences of both enzymes, their different patterns of genetic association with various diseases, the role of polymorphisms determining changes in enzymatic activity or expression levels, and the distinct peptidomes of disease-associated MHC-I allotypes. ERAP1 and ERAP2 polymorphism and expression induce significant changes in multiple MHC-I-bound peptidomes. These changes are MHC allotype-specific and, without excluding a degree of functional inter-dependence between both enzymes, reflect largely separate roles in their processing of MHC-I ligands. The studies reviewed here provide a molecular basis for the distinct patterns of genetic association of ERAP1 and ERAP2 with disease and for the pathogenetic role of peptides. The allotype-dependent alterations induced on distinct peptidomes may explain that the joint association of both enzymes and unrelated MHC-I alleles influence different pathological outcomes.

## Introduction

The role of the Major Histocompatibility Complex (MHC) as a risk factor for numerous diseases is well established (1). Although usually involving MHC-II alleles a more restricted set of inflammatory disorders is strongly associated with MHC-I. The most prominent of these associations concern birdshot chorioretinopathy (BSCR), ankylosing spondylitis (AS), Behçet's disease (BD) and psoriasis, which involve HLA-A^*^29:02, HLA-B^*^27, HLA-B^*^51, and HLA-C^*^06:02, respectively (2–10). The associations of BSCR and AS with MHC-I are extremely strong, with about 100 and 90% of patients being A^*^29:02 and B^*^27 positive, respectively. The associations of HLA-B^*^51 and C^*^06:02 with BD and psoriasis are lower, yet quite significant, with odds ratios around 5–6.

In spite of the strength of these associations, both the pathogenetic mechanisms and the role of the MHC molecules in the four diseases remain unknown. Whereas, BSCR is an eye-specific inflammatory disorder, typically affecting the choroid and retina (11), the three other diseases show distinct systemic manifestations, involving a variety of tissues, cell populations, and immunological pathways, a complexity that poses considerable difficulty to the characterization of pathogenetic factors (12–14).

The development of genome wide association studies and other genetic strategies lead, among many other findings, to a discovery that promised to shed new light into the pathogenetic role of MHC-I in disease. Namely, the involvement of the endoplasmic reticulum aminopeptidases (ERAP)1 and 2 as prominent risk factors for MHC-I-associated diseases (12). These enzymes are key players in the antigen processing pathway of MHC-I molecules because they trim peptides to the optimal size for MHC-I binding (15, 16) and can also over-trim and destroy MHC-I ligands (17–20).

ERAP1 is associated with AS, psoriasis and BD in epistasis with the risk HLA-B^*^27 (21) and -B^*^40:01 (22), C^*^06:02, (23), and HLA-B^*^51 alleles (24), respectively. ERAP2 is associated with AS and psoriasis (25–28), but not in epistasis with MHC-I. ERAP2 and ERAP1 are also risk factors for BSCR (2, 29).

The joint association of ERAP1/2 and MHC-I with multiple inflammatory disorders underlines the relevance of MHC-I-bound peptides in these diseases. Since peptides presented by MHC-I determine both the recognition by T lymphocytes (30, 31) and NK cells (32), ERAP1 and ERAP2 can influence both innate and adaptive immune pathways in these diseases. Indeed, a role of ERAP1 in modulating innate immunity has been demonstrated (33, 34), and its involvement in cross-presentation has also been claimed (35–37).

ERAP1, and perhaps also ERAP2, may have additional functions besides antigen processing (38, 39). For instance, ERAP1 can be secreted from macrophages in response to lipopolyssacharide and interferon-gamma by a TLR-dependent mechanism (40), which enhances their phagocytic activity (41). This might be relevant to the modulation of innate immunity. A potentially important issue is the involvement of both ERAP1 and ERAP2 in the blood pressure homeostasis, as revealed by genetic analyses (42, 43). The relevance of secreted ERAP1 in this process is supported by a study showing that the redox state of the ER-resident thioredoxin ERp44 controls blood pressure. This protein is involved in the retention of ERAP1 and other proteins in the ER by a thiol-dependent mechanism (44). ERAP2 secretion might be promoted in an analogous way but, to my knowledge, this has not been demonstrated. The mechanism by which secreted ERAP1 and ERAP2 may modulate hypertension is not yet resolved. These enzymes may influence the processing of relevant peptide hormones regulating blood pressure (44–46). In addition, cleavage of Arg residues by secreted enzymes, may promote the formation of nitric oxide, as shown for ERAP1 (47), which may in turn influence blood pressure and inflammation (48). The putative relevance of these alternative functions should not be underestimated. Yet, it seems that the interplay between ERAP1/ERAP2 and MHC-I molecules should hide the mechanism underlying their joint association with disease.

This review will focus on recent studies dealing with the effects of the polymorphism and expression of ERAP1 and ERAP2 on shaping the constitutive peptidomes of disease-associated MHC-I proteins from live cells, and will discuss the contribution of these studies to our understanding of ERAP1/ERAP2 function and pathogenetic role.

## ERAP1 and ERAP2 have complementary substrate preferences

ERAP1 and ERAP2 are structurally related Zn-metallopeptidases that share about 50% amino acid sequence identity and many similarities in their three-dimensional structure (49–52), reviewed in Stratikos and Stern (53). Both enzymes consist in four globular domains. Their catalytic sites are located in domain II and a cavity, encompassing areas of domains II and IV, is the substrate-binding site. Conformational changes involving the reorientation of amino acid side chains in the catalytic site and domain rearrangements leading to open (less active) and closed (more active) conformations govern the enzymatic activity of ERAP1 and, probably, of ERAP2. Yet, the distinct structure of both the active site and the peptide binding site determine critical differences in enzymatic specificity and substrate handling. For the purpose of this review, the most important of these differences are those concerning N-terminal residue specificity and substrate length preferences. ERAP1 can cleave all peptide bonds except those involving Pro, but shows a wide range of efficiency depending on the N-terminal side chain of the substrate: it shows preference for nonpolar residues and is much less efficient with polar and charged ones (54). ERAP2 can cleave very few residues, being most efficient with Arg (12, 16, 45). In addition, ERAP1 preferentially cleaves peptides longer than 9-mers and becomes virtually inactive with 8-mers and shorter peptides. This remarkable feature, which is seemingly unique among aminopeptidases, is known as the *molecular ruler* mechanism (55) and tunes the length preferences of the enzyme to the optimal length of MHC-I ligands. In contrast, ERAP2 cleaves best 9-mers and shorter peptides, becoming progressively less efficient with longer ones (52).

## ERAP1 polymorphism and expression

ERAP1 is expressed in all individuals and displays a significant degree of polymorphism, much of which affects the enzymatic activity, the expression level or both. Natural ERAP1 variants are complex allotypes including multiple non-synonymous single nucleotide polymorphisms (SNPs), known as haplotypes. Ten ERAP1 haplotypes, designated as Hap1 to Hap10 account for over 99% of the natural ERAP1 variants in human populations (56). Polymorphic amino acids are frequently located near the catalytic site (residues 346, 349), in the peptide binding site (residues 725 and 730) or in interdomain regions or other locations that can affect the conformational rearrangements associated with the acquisition of the enzymatic activity (residues 528 and 575). Therefore, these polymorphisms may influence ERAP1 activity in multiple ways. Several *in vitro* studies have established that the K528R polymorphism has large effects (19–21, 46, 57), due to its influence on the kinetics of the conformational transition between the active and inactive states of the enzyme (58). In contrast, the Q730E change influences the enzymatic activity in a way that depends on substrate length, by increasing the preference of ERAP1 for shorter substrates (58). This is likely related to the involvement of this residue in substrate binding.

The activity of natural ERAP1 variants depends on the specific combination of polymorphic residues (19, 20) and, due to the diverse alterations induced by different mutations, the activity of a given variant cannot be easily predicted from the effects of single changes. Yet, natural ERAP1 variants with K528 (Hap1–Hap3) are more active than those with R528, and the less active haplotype, Hap10, is the one that accumulates the largest number of amino changes that negatively affect ERAP1 activity.

Other ERAP1 polymorphisms affect the expression level, rather than the structure, of ERAP1, as well as the relative levels of the two isoforms of this enzyme commonly expressed in all individuals (59–61). Whereas, differences in ERAP1 expression have an obvious effect on peptide cleavage, the effect of altered isoform levels is less straightforward. It seems that differences in the posttranscriptional dynamics between the two isoforms results in an indirect influence of the polymorphism affecting isoform proportions on the overall expression of the ERAP1 protein (61).

Due to the complexity and variety of effects induced by ERAP1 polymorphism, the functional spectrum of this enzyme across individuals is potentially very diverse. Yet, tight linkage disequilibrium (LD) among many of these polymorphisms probably limits this diversity at the population level. LD can also increase the effect of single SNPs on enzymatic activity by promoting higher expression of the more active allotype. A paradigmatic case is the correlation between the highly active variant K528 with SNPs determining increased ERAP1 expression (60–62).

## ERAP2 polymorphism and expression

In contrast to ERAP1, non-synonymous changes affecting the amino acid sequence of ERAP2 seem to be very limited. A polymorphism coding for the K392N change affects ERAP2 activity (63). Although both variants are expressed in some populations (64), the N392 allele is in strong LD with SNP rs2248374, a frequent polymorphism that promotes nonsense-mediated RNA decay and impairs protein expression (65). As a result, only the K392 variant is expressed in most populations. Since both rs2248374 alleles show similar frequencies due to balancing selection about 25% of individuals fail to express ERAP2. Other polymorphisms, such as rs10044354, or SNPs in strong LD with it, also have a large impact on ERAP2 protein expression (2, 66). Heterozygosity at these loci, and probably additional polymorphisms influencing quantitative expression of the enzyme (61), introduce a further level of heterogeneity in the activity of ERAP2 across individuals due to different enzyme levels.

In a recent study a polymorphism located in the ERAP2 promoter region, rs7586269, was associated with opposite changes in the expression of ERAP1 and ERAP2, so that decreased expression of ERAP2 correlated with increased ERAP1 expression, suggesting a concerted regulation of the expression of both genes by this SNP (67). Yet, unlike rs2248374 and rs10044354, the minor allele frequency of rs7586269 is rather low in most populations.

## Risk and protective ERAP1 and ERAP2 polymorphisms in MHC-I-associated diseases

### Ankylosing spondylitis

Although a number of non-synonymous ERAP1 polymorphisms were initially found to influence risk of AS (60, 68), the strong LD within the ERAP1 gene required further refinement to dissect the actual disease-promoting polymorphisms. The association of ERAP1 with AS was most consistent with a two-mutation model involving a primary effect of the high activity K528 variant and a secondary effect determined by D575/R725 (21). Both polymorphisms are jointly present in the AS-associated Hap1 to Hap3 haplotypes (56). Accordingly, Hap10, a low activity haplotype including the two protective changes R528 and N575/Q725, significantly influenced AS protection. The Q730E polymorphism showed a strong association with AS, Q730 being the risk allotype, but fine mapping studies narrowed down the AS-promoting region of ERAP1 to a portion of the gene that excludes codon 730 (69), raising the possibility that Q730E might not directly influence AS risk. In addition, recent analyses suggest that the association of D575/R725, tagged by rs10050860 at codon 575, is in tight LD with the splice altering SNP rs7063, which is the one that seems to account for the contribution to AS susceptibility (61). The risk allele of this polymorphism leads to increased expression of the shortest ERAP1 isoform, 19E, and higher ERAP1 protein levels. Thus, our current view is that AS risk is favored by high ERAP1 activity, mainly due to the direct effect of K528, without excluding some additional effect of D575/R725, and to linked polymorphisms increasing ERAP1 protein expression of the high activity variants.

ERAP2 expression, as determined by the rs2248374 SNP, is associated with AS risk, both in HLA-B^*^27-positive and -negative individuals (26). One or more additional polymorphisms in tight LD additionally influence ERAP2 expression in a quantitative way. The corresponding risk alleles are those determining increased ERAP2 levels (61). Thus, as for ERAP1, AS risk is favored by ERAP2 activity.

### Behçet's disease

The initial report detecting the epistatic association of ERAP1 and HLA-B^*^51 with BD showed that the polymorphisms encoding the D575N/R725Q changes were associated with disease risk, fitting a recessive model, so that risk of BD is conferred by homozygosity at this locus (24). Further studies determined that Hap10 is the risk haplotype for this disease (70). Thus, the same ERAP1 variant that is protective for AS increases risk of BD, acting in both cases in epistasis with the susceptibility MHC allele. This finding strongly suggests that the pathogenetic role of ERAP1 in the two diseases is MHC-I allotype-dependent, presumably related to effects on the processing of their corresponding ligands.

To my knowledge there is no evidence for an association of ERAP2 with BD, making this disease the only one of the four major MHC-I associated disorders for which a role of this enzyme has not been demonstrated.

### Birdshot chorioretinopathy

Evidence for an involvement of ERAP1 in BSCR was suspected from the significant effects of ERAP1 polymorphism on the A^*^29:02 peptidome (66) and has been demonstrated in a recent study (29) that identified the low activity Hap10 haplotype as a risk factor for this disease. Its contribution was further enhanced by co-occurrence with the splice-altering variant rs7063, influencing the relative expression of the ERAP1 isoforms, whose risk allele (T) determines lower protein levels. Unlike the situation in BD, the association of Hap10 with BSCR does not appear to follow a recessive model. Also unlike BD, ERAP2 presence and higher expression level are risk factors for BSCR (2, 29).

### Psoriasis

The association of ERAP1 with psoriasis shows a similar pattern as in AS: multiple studies, reviewed in Lopez de Castro et al. (12) and Ombrello et al. (56), suggest that K528 and Q730 in ERAP1 predispose to disease in epistasis with C^*^06:02. The major risk haplotype is Hap2, which includes these two residues, and the protective haplotype is Hap10, which includes residues R528 and E730. The epistasis between ERAP1 and C^*^06:02 was challenged by one study upon stratification of patients by age at onset (71).

Defining the association of ERAP2 with psoriasis (27, 28) has been complicated due to the seemingly non-epistatic nature of this association, the heterogeneity of the disease and the apparently opposite effects of ERAP1 and ERAP2 in the same haplotype, leading to a masking of the latter by ERAP1. Conditional analyses controlling for the contribution of ERAP1 suggested an association of ERAP2 with psoriasis independent of ERAP1 (27) and also that the risk allele of ERAP1 and the protective allele of ERAP2, the latter tagged by the intronic SNP rs2910686, frequently segregated together. Since the risk allele of rs2910686 (C) was the same as in AS (25) this result suggests that, like in AS, ERAP2 expression may predispose to psoriasis, although the effect may often be masked by ERAP1. In these studies no subdivision of disease by association with C^*^06:02 or age at onset was considered. In a recent report from Poland (72) such subdivision was carried out. Although somewhat limited by the size of the cohorts, this study, which tagged ERAP2 using rs2248374, a polymorphism directly determining ERAP2 protein expression (65), found that presence of ERAP2 was protective from psoriasis, although this association showed up only among C^*^06:02-positive individuals with very early disease onset. In spite of the limitations imposed by sample size, this and other studies suggest that the association of both ERAP1 and ERAP2 with psoriasis is probably variable in this heterogeneous pathology, and a precise definition of the role of these enzymes, and their functional interaction with C^*^06:02, should benefit from large scale studies allowing robust statistical assessments within disease subsets.

The influence of ERAP1 and ERAP2 in MHC-I-associated diseases is summarized in Table 1.

**Table 1:** ERAP1 and ERAP2 in MHC-I-associated diseases^a^.

**Disease**	**ERAP1 risk**^**b**^		**ERAP2 risk**	
	**Genetics**	**Enzymatic activity**	**Genetics**	**Enzymatic activity**
Ankylosing spondylitis	Hap1-Hap3 Higher expression	High	ERAP2 presence and higher expression level	High
Behçet's disease	Hap10 (recessive)	Low	Not reported	Not reported
Birdshot chorioretinopathy	Hap10 (non-recessive) Lower expression	Low	ERAP2 presence and higher expression level	High
Psoriasis	Hap2 Disease subset dependent (?)	High Disease subset dependent (?)	Disease subset dependent (?)	Disease subset dependent (?)

a *See text for references*.

b ERAP1 is in epistasis with the susceptibility MHC-I allele at least in AS, BD, and psoriasis, although in the latter might be differences among disease subsets. As for BSCR, the association with A^*^29:02 is virtually 100% (3). For the polymorphic nucleotide and amino acid changes of the Hap1-Hap3 and Hap10 variants of ERAP1 see (56).

## The peptidomes of disease associated MHC-I molecules

**Figure 1:**
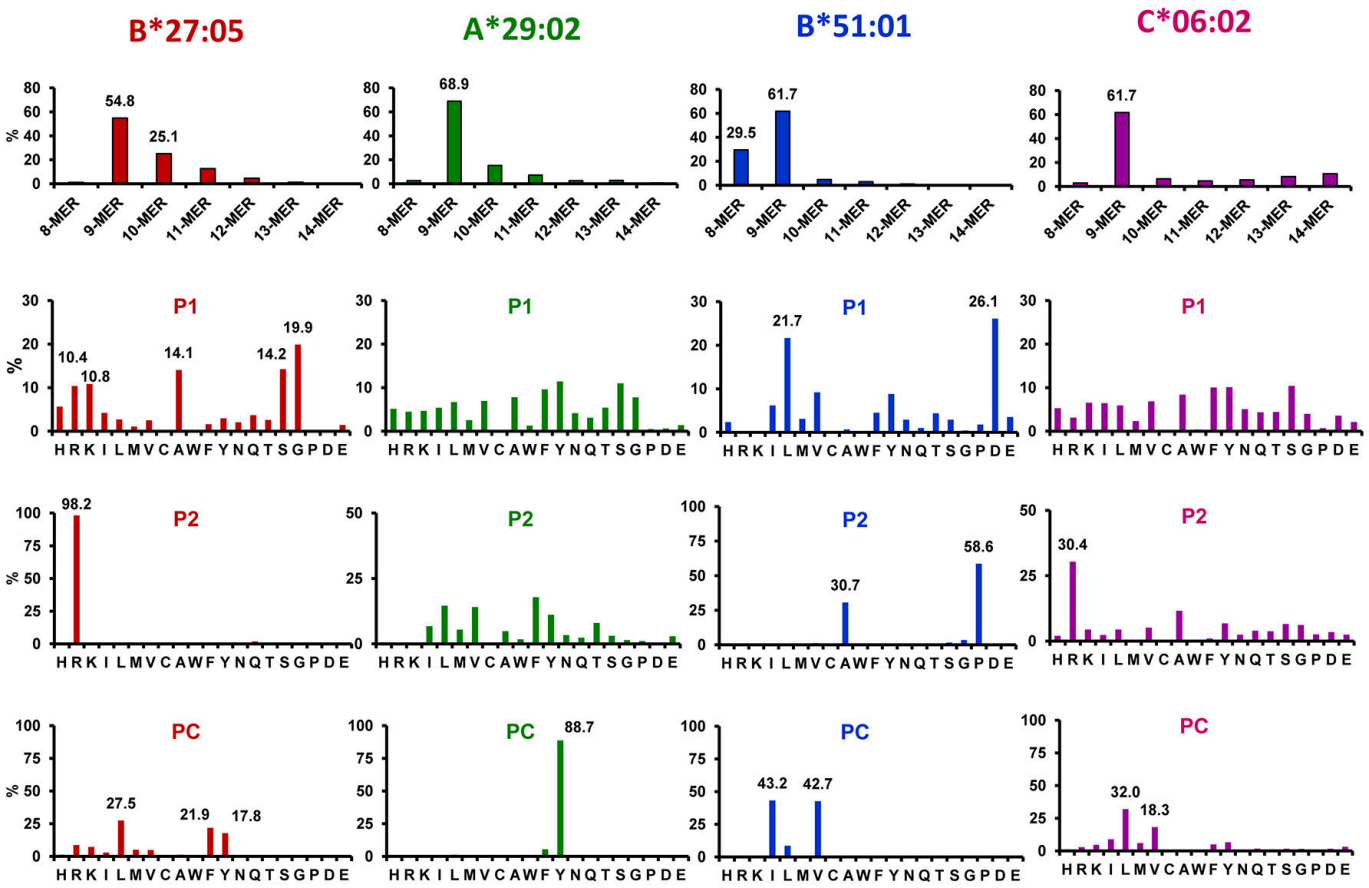
Major features of the peptidomes of disease-associated MHC-I molecules. Length distribution and % residue frequencies at P1, P2, and PC of B*27:05, A*29:02, B*51:01, and C*06:02 ligands (N: 1,117, 4,931, 1,620, and 1,287 peptides, respectively). The data are from the following references: B*27:05 (79), A*29:02 (66), B*51:01 (80), and C*06:02 (81).

As virtually all other human MHC-I molecules, the peptidomes of those associated with disease share a number of fundamental features, the most important of which are the following (73–78): (1) MHC-I ligands show strong length restrictions, with an optimal size of nine amino acid residues, and frequencies falling abruptly with shorter or larger length, (2) peptides are bound to the MHC-I molecule into specific pockets of its peptide binding site, termed A–F; the main interactions involve the peptidic amino- and carboxyl-termini, in pockets A and F, respectively, as well as a few main anchor residues, which in humans are generally located at position (P)2 and C-terminal (PC) and bind in pockets B and F, respectively, (3) other interactions involve the peptidic main chain and a number of additional amino acid side chains of secondary anchor residues, which are generally those at P1, P3, and PC-2, without excluding others. Different HLA-A, -B, and -C allelic products typically differ in the nature of the main anchor residues, which provide the optimal binding to each particular allotype. MHC-I polymorphism can dramatically alter the size, shape, and polarity of side chain binding pockets. The main features of the ligands of major disease-associated MHC-I molecules are summarized in Figure 1.

### The HLA-B^*^27 peptidome

HLA-B^*^27 was one of the earliest MHC molecules whose structure (76, 77) and peptidome (82) were investigated, and one of the most intensively studied, due to its association with AS. The initial studies already determined that the main anchor residues of the B^*^27:05 prototype were Arg2 and basic, aliphatic and aromatic residues at PC. Whereas, Arg2 was a hallmark of most HLA-B^*^27 ligands, the polymorphism of the major subtypes, including some differentially associated with AS, frequently affected C-terminal residues, drastically decreasing the tolerance for basic residues (i.e., B^*^27:02, B^*^27:04), or restricting this position almost only to nonpolar ones (i.e., B^*^27:06) (83). With the arrival of mass spectrometry-based immunopeptidomics, the identification of high numbers of HLA-B^*^27 ligands allowed the refinement of these motifs and the characterization of new ones (79, 84–86), providing a much more accurate picture of the HLA-B^*^27 peptidome that, nevertheless did not alter the essential features defined by earlier studies. One of these features, which is particularly relevant to this review, is the remarkable preference of HLA-B27 for basic (R, K) and small (G, A, S) N-terminal residues, which account for about 20 and 48% of the peptidome, respectively. Along with the Arg2 motif, the high frequency of HLA-B^*^27 ligands with dibasic N-terminal sequences, which are particularly resistant to digestion by cytosolic amino-peptidases, might endow HLA-B^*^27 with the capacity to present peptides generated in particularly low amounts (87). In addition, the HLA-B^*^27 binding motif is particularly well suited to the TAP binding preferences (88–90), which should favor the import into the ER of HLA-B^*^27 ligands directly generated in the cytosol.

### The HLA-A^*^29:02 peptidome

Beyond earlier studies (91), the most extensive characterization of the A^*^29:02 peptidome is found in three recent reports (66, 92, 93). Unlike other disease-associated MHC-I molecules A^*^29:02 shows only one dominant, highly restrictive, main anchor motif of Tyr at PC. Some looser restriction for aliphatic/aromatic residues is observed at P2 and PC-2. There are no clear preferences at P1, although aromatic residues (Y, F) jointly account for about 20% of the A^*^29:02 ligands.

### The HLA-B^*^51 peptidome

Several distinctive features, compared to other disease-associated MHC-I molecules, characterize the HLA-B^*^51 peptidome (80, 92). It shows a particularly high percentage of octamers and low allowance for peptides >9-mers. Most important, it has a dual motif of Ala2 and Pro2, which defines two very different subpeptidomes, both of which share a same, very restrictive, C-terminal motif of aliphatic residues, including Val and Ile, and a lower percentage of Leu. The Ala2 and Pro2 subpeptidomes drastically differ in the usage of P1 residues. Whereas, Leu and other nonpolar residues are favored at P1 among peptides with Pro2, in the Ala2 subpeptidome Asp1 is largely predominant. The Ala2 subpeptidome shows lower affinity (80), which is consistent with experimental binding studies (94, 95). Uniquely among de major disease-associated MHC-I molecules, basic residues at P1 are virtually absent among HLA-B^*^51 ligands, a feature that might be related to the lack of association of ERAP2 with BD.

### The HLA-C^*^06:02 peptidome

By far the best characterization of the C^*^06:02 peptidome has been reported by Mobbs and colleagues (81). Based on their study this molecule binds mainly 9-mers, is less tolerant than HLA-B27 for 10-mers and 11-mers, and somewhat more for longer peptides. The predominant residue at P2 is Arg, but its frequency is much smaller than in HLA-B^*^27 (about 30 vs. >95%). C-terminal residues are predominantly aliphatic/aromatic, which is frequent among MHC-I molecules, including HLA-B^*^27. A major distinctive feature of C^*^06:02 is a prominent motif of basic residues at PC-2 (R+K: ~41%), suggesting that this may be a main anchor position of C^*^06:02 ligands. For the purpose of this review it is important to note the diversity at P1, which is comparable to that in the A^*^29:02 peptidome, so that no single residue has a frequency higher than ≈10%.

## ERAP1 and the peptidomes of disease-associated MHC-I molecules

Two types of studies have addressed the role of ERAP1 on shaping disease-associated MHC-I peptidomes: those involving the depletion of ERAP1 and those addressing the role of ERAP1 polymorphism. ERAP1 is expressed in the normal cells of all individuals, although its expression can be largely diminished or absent altogether in transformed cells (96). The effects of ERAP1 downregulation on the peptidome have been examined so far in HLA-B^*^27 (97, 98) and A^*^29:02 (66).The effects of ERAP1 polymorphism on the peptidomes expressed in live cells have been examined in HLA-B^*^27 (99–102), HLA-A^*^29 (66), and HLA-B^*^51 (103).

### ERAP1 and the HLA-B^*^27 peptidome

#### Effects of ERAP1 depletion

Depletion of ERAP1 in human cells in one study (97) resulted in longer peptides, a significant increase of C-terminally extended ligands, and some alteration in P1 residue frequencies. The percentage of 9-mers relative to longer peptides decreased from about 50% in the wild type to about 35% in the ERAP1-inhibited cell line. An 8-fold increase of extended ligands (from 3 to 24 peptides) was observed in ERAP1-inhibited cells compared to the ERAP1 proficient counterpart, of which most were C-terminally extended peptides carrying the Arg2 motif. These observations illustrate the relevance of ERAP1 in optimizing the length of MHC-I ligands, a well-established function of this enzyme. Some fluctuation in P1 residue frequencies, including a decrease of Arg1, was also observed in ERAP1-deficient cells. However, the moderate number of identified peptides in this study and the low frequency of many P1 residues limited the statistical significance of these fluctuations and precluded a precise assessment of the influence of ERAP1 on P1 residue usage.

A more recent study reported a huge number (about 15,000) of HLA-B^*^27 ligands from ERAP1-proficient transgenic rats and the counterparts carrying a homozygous deletion of the ERAP1 gene (98). An interesting outcome of this study, also observed with functional ERAP1 variants (see below) is that only about one-third of the HLA-B^*^27 peptidome was altered upon ERAP1 depletion, suggesting that most of the peptides are not subjected to ERAP1 processing or that their generation/destruction balance results in unchanged peptide amounts, relative to absence of the enzyme. A significant effect on peptide length, resulting in longer ligands in the absence of ERAP1 was also observed, as well as changes in P1 residue usage, in particular among peptides showing quantitative expression differences. For instance, Gly1 was more frequent among peptides predominant in the presence of ERAP1 and Ala1, Ser1, and Lys1 were increased in the absence of the enzyme. Another interesting observation is that the main anchor residues that most contribute to binding affinity, Arg2 and C-terminal aliphatic/aromatic residues, were more frequent among peptides longer tan 9-mers, which suggests a compensatory effect for their suboptimal length.

#### Effects of ERAP1 polymorphism

In various studies from our laboratory (99–102) we addressed the effects of natural ERAP1 polymorphism on the HLA-B^*^27 peptidome. The focus was on the quantitative changes in peptide amounts, rather than on peptides detected only in a specific ERAP1 context, and the strategy used was to compare the HLA-B^*^27 peptidomes from human cell lines expressing distinct ERAP1 variants. Already in our initial study (99), we observed that the HLA-B^*^27 peptidome expressed in the context of an AS-associated ERAP1 haplotype (Hap2) differed substantially from the peptidome expressed in an AS-protective context (Hap10) in showing a higher percentage of 9-mers and increased frequencies of ERAP1-resistant flanking and P1 residues. A second study (100) further determined that of the two polymorphisms accounting for the association of ERAP1 with AS, namely at residues 528 and 575/725, the former one dominated the alterations of the HLA-B^*^27 peptidome.

**Figure 2:**
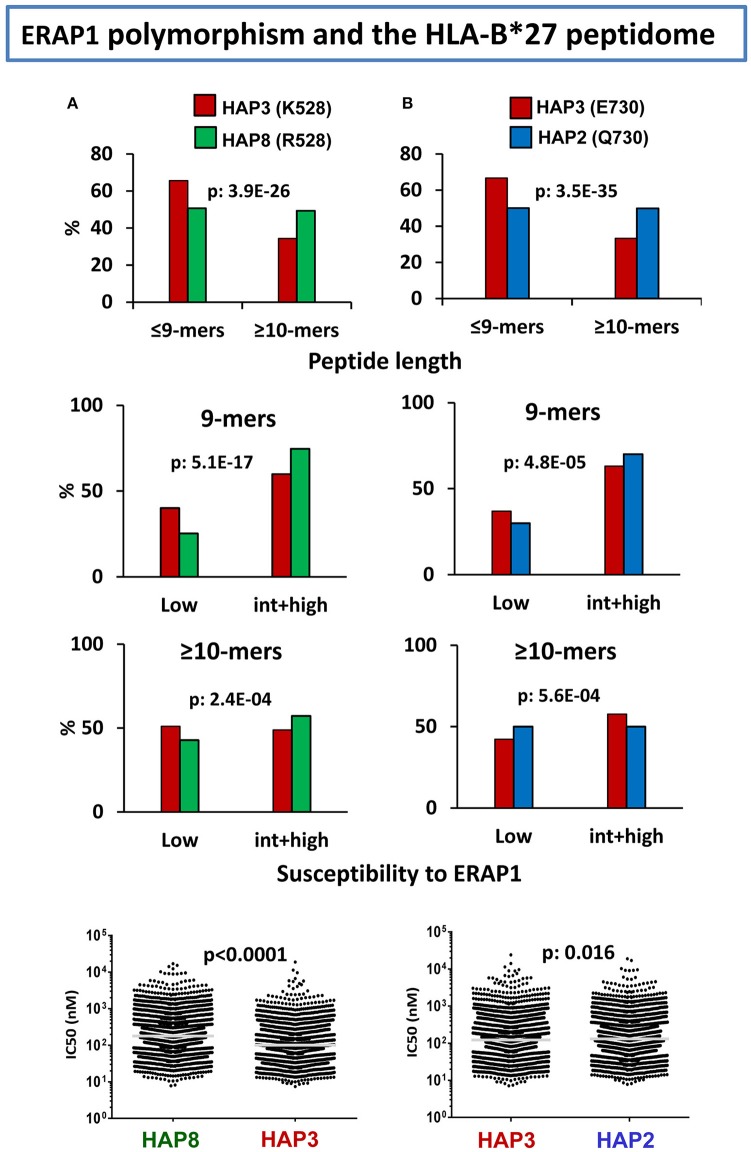
Influence of ERAP1 polymorphism on the HLA-B*27 peptidome. **(A)** Effects of K528R. Comparison of the B*27:05 ligands over-represented (>1-fold) in a cell line (LG2, *N*: 2215 peptides) expressing the Hap3 variant of ERAP1 (red), with those over-represented in a cell line (C1R05, N: 2801) expressing the Hap8 variant (green). Hap3 and Hap8 differ by R127P, I276M, and K528R, but the two former changes have much less influence on peptide trimming. The comparisons involve peptide length (upper panel), joint frequencies of P1 residues with low or intermediate+high susceptibility to ERAP1 among 9-mers (second panel) or longer peptides (third panel), and theoretical affinity of the total ligands, with the medians indicated by bars. **(B)** Effects of E730Q. Comparison of the B*27:05 ligands over-represented (>1-fold) in the LG2 cell line (Hap3: red), with those over-represented in LCL 6370 (blue) expressing the Hap2 variant (N: 2,444 and 2,869 peptides, respectively). Hap3 and Hap2 differ only by the E730Q change. Conventions are as in **(A)**. The statistical significance of the changes is indicated by the *p*-values, as estimated by the χ^2^ (three upper panels) or Mann–Whitney tests (lower panels). These data were originally published in reference (102).

Considering the complexity of ERAP1 haplotypes, the diverse influence of individual mutations (58), and the combined effects of co-occurring polymorphisms (19, 20), we recently attempted to rank the effects of individual changes in ERAP1 on the HLA-B^*^27 peptidome through a multilevel comparison between cell lines with appropriate ERAP1 backgrounds (102). This study confirmed the dominant effect of K528R on decreasing the enzymatic activity of ERAP1, as reflected mainly in the increased frequency of 9-mers, relative to longer peptides, the skewing of P1 frequencies toward ERAP1-resistant residues, and the increased affinity of the peptidome in the K528 context (Figure 2A). The effect of D575N/R725Q was much smaller, but added to that of polymorphism at residue 528 in decreasing ERAP1 trimming, thus accounting for the low activity of the protective Hap10 haplotype, carrying both changes. This study also showed a notorious effect of the Q730E polymorphism on trimming depending on peptide length, which had been previously noted *in vitro* using poly-Gly analogs (58). In HLA-B^*^27 this was reflected in higher abundance of shorter peptides (≤9-mers) with E730, and opposite effects of the E730Q change on P1 residue frequencies among 9-mers compared to longer peptides, depending on their susceptibility to ERAP1 trimming (Figure 2B), with little consequence on the global affinity of the peptidome. The distinct alterations resulting from the length-independent effects of K528R and the length-dependent effects of Q730E introduce a significant complexity in the functional features of ERAP1 haplotypes. Thus, at the population level, ERAP1 polymorphism can influence the HLA-B27 peptidome in very diverse ways, due to the multiplicity of ERAP1 haplotypes and the distinct combinations of functionally different mutations within each haplotype.

Further complexity is introduced by polymorphisms that affect ERAP1 protein levels, either by altering gene expression or through alternative splicing (61). For instance, the risk K528 variant of ERAP1 is consistently associated with higher ERAP1 protein because the SNP rs30187, coding for the K528R change, is in LD with one or more polymorphisms altering gene expression (60–62). In addition, as already mentioned, the splicing altering variant rs7603 determines the relative amounts of two ERAP1 isoforms differing in Exon 20, which codes for a short C-terminal portion of the molecule. Because of their distinct transcriptional dynamics, alterations in the relative expression of both isoforms result in distinct protein levels. Conditional analyses showed that rs7603 accounted for the association of D575N/R725Q with AS, suggesting that the contribution of this protein polymorphism to disease risk is mainly through the effect of the linked rs7603 allele on increasing protein levels (61). This may explain the significant association of D575N/R725Q with AS in spite of the relatively minor effect of these changes on the B^*^27:05 peptidome (100, 102).

### ERAP1 and the HLA-A^*^29:02 peptidome

**Figure 3:**
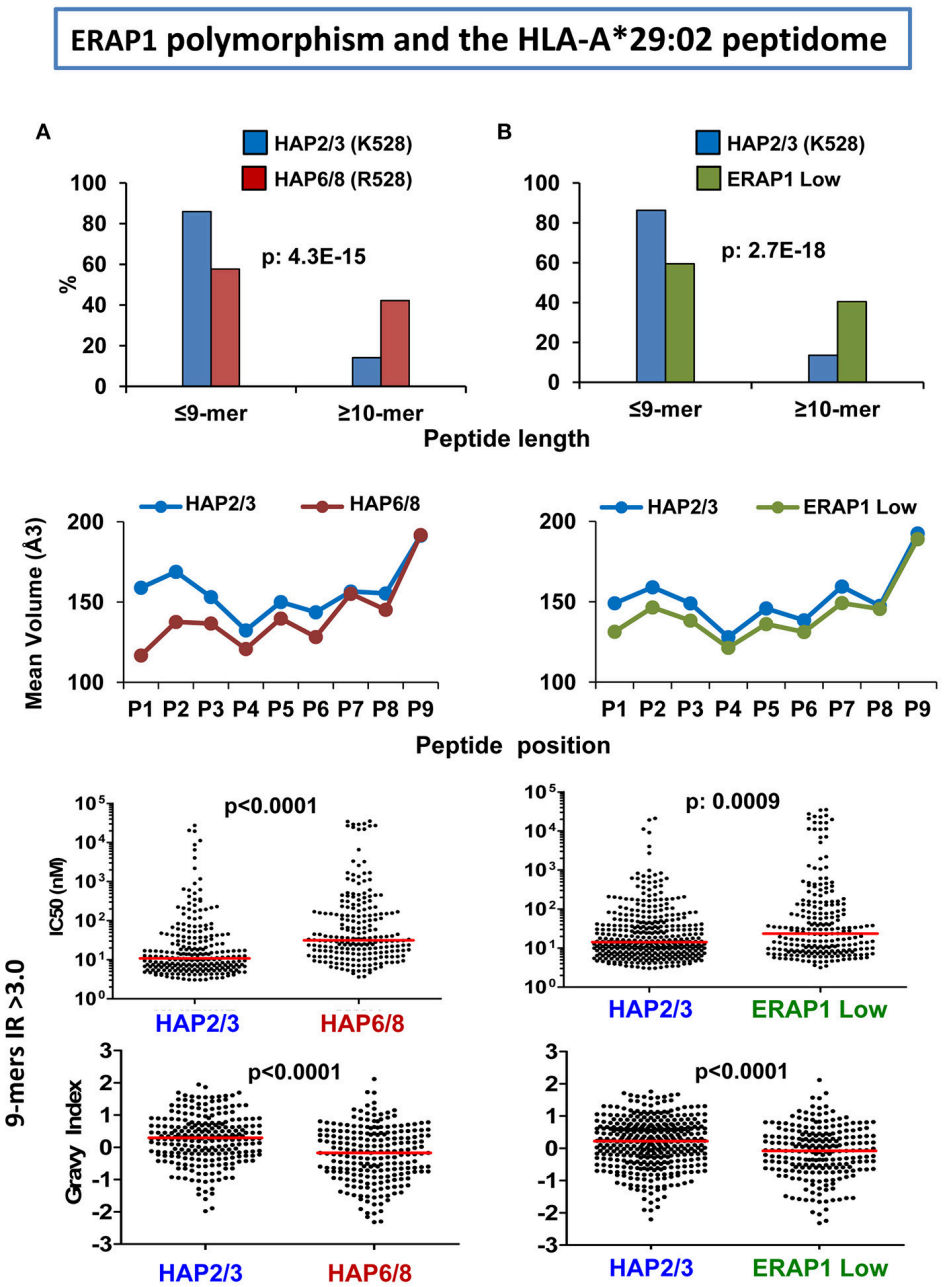
Influence of ERAP1 on the A*29:02 peptidome. **(A)** Comparison of the A*29:02 ligands over-represented (>3-fold) in a cell line (PF97387, N: 292 peptides) expressing the Hap2/Hap3 variants of ERAP1 (blue), with those equally over-represented in a cell line (MOU, N: 383) expressing the Hap6/Hap8 variants (red). Both haplotype combinations differ by the R127P and K528R changes. The comparisons involve peptide length (upper panel), mean side chain volume at each peptide position among the 9-mers in these peptide sets (second panel), theoretical affinity of these 9-mers, with the medians indicated by bars (third panel), and hydropathy of the same peptides, estimated by the Grand Average of Hydropathy (GRAVY) index (lower panel). **(B)** Comparison of the A*29:02 ligands over-represented (>3-fold) in PF97387 (*N*: 446 peptides), with those equally over-represented in the cell line SWEIG (N: 390 peptides) expressing very low levels of ERAP1 (green). All conventions are as in **(A)**. The statistical significance of the changes is indicated by the *p*-values, as estimated by the χ^2^ (upper panels) or Mann–Whitney tests (lower panels). These data were originally published in reference (66).

The effects of ERAP1 on the A^*^29:02 peptidome were analyzed through comparisons involving three ERAP2-negative LCL, two of which expressed ERAP1 variants with distinct enzymatic activity, and the third one expressing very low ERAP1 levels (66). In a high activity ERAP1 context (Hap2/3) the A^*^29:02 peptidome showed the following differences compared to either a peptidome expressed in the context of lower activity variants (Hap6/8) or low ERAP1 protein levels (about 16-fold less): (1) increased amounts of 9-mers relative to longer peptides, (2) bulkier and more hydrophobic P1 and P2 residues, (3) higher global affinity for A^*^29:02, (4) higher global hydrophobicity (Figure 3). These effects were similar in both comparisons, indicating rather small differences between the A^*^29:02 peptidomes generated with either low activity ERAP1 variants or low ERAP1 levels. Like in HLA-B^*^27 these changes affected only a fraction of the peptidome and the amounts of many ligands remained essentially unaltered regardless of the ERAP1 context. Given the association of the low activity Hap10 haplotype with BSCR (29) this disease might be favored by an A^*^29:02 peptidome with at least a fraction of peptides showing suboptimal length, lower hydrophobicity and decreased affinity.

### ERAP1 and the HLA-B^*^51 peptidome

Our initial characterization of the HLA-B^*^51 peptidome (80) concerned B^*^51:01 in an ERAP2-positive cell line (721.221) expressing the Hap1/Hap8 haplotypes (104), of which Hap1 is of high activity. In another study we determined the features of the B^*^51:08 peptidome from an ERAP2-positive cell line expressing the low activity ERAP1 variant Hap10 (103). B51:08 is, like B^*^51:01, associated with BD and differs from the latter only by the E152V and L156D changes (103, 105). Hap10 is the ERAP1 haplotype associated with risk of BD (70). Therefore, it was of interest to assess the effect of this low activity haplotype on the HLA-B^*^51 peptidome. The location of the polymorphic residues between B^*^51:01 and B^*^51:08 at positions far away from the interaction site of the peptidic N-terminus facilitated the distinction of the effects due to subtype polymorphism from those depending on the ERAP1 context when comparing both HLA-B^*^51 peptidomes (103).

**Figure 4:**
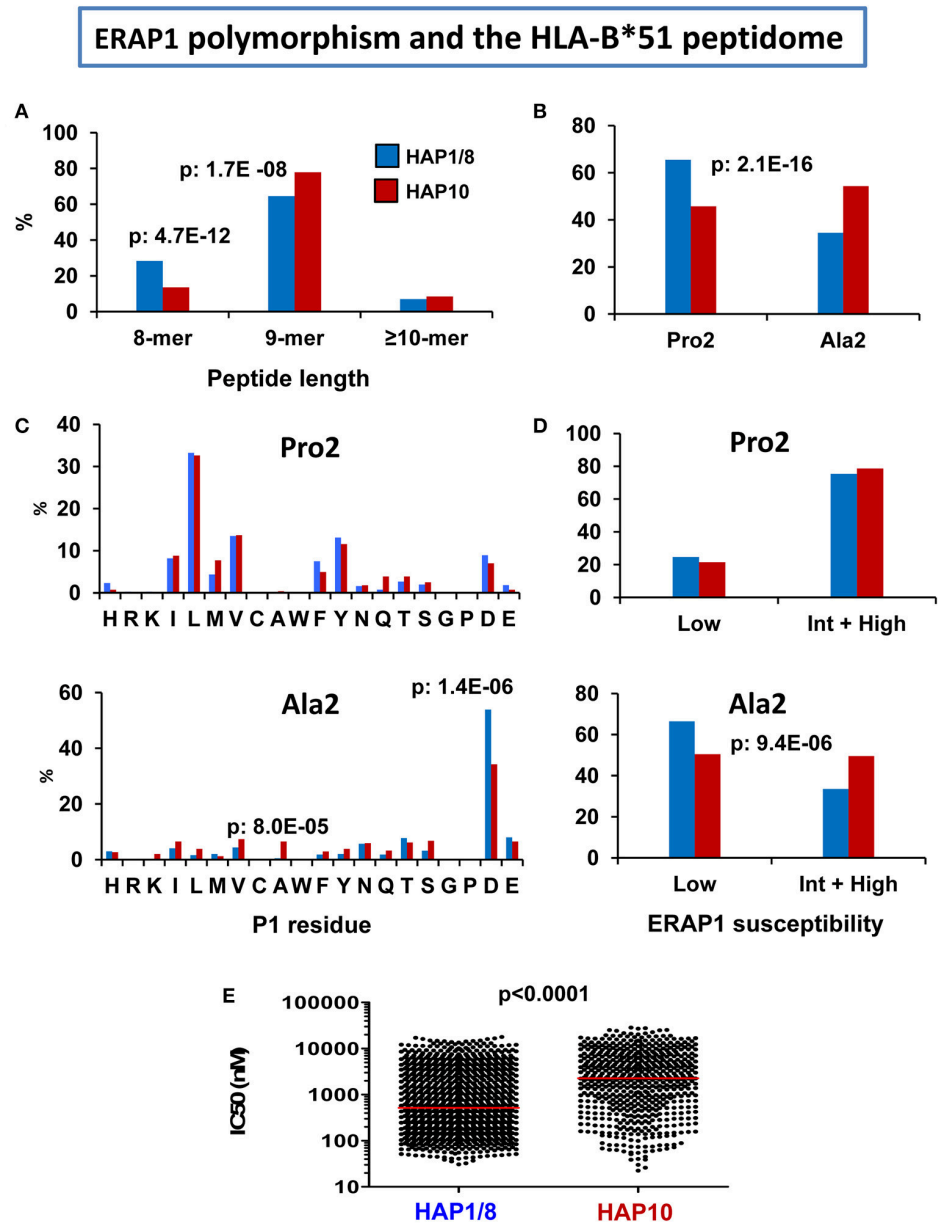
Influence of ERAP1 polymorphism on the HLA-B*51 peptidome. Comparison of the HLA-B*51:01 ligands from the transfectant cell line 721.221-B*51 (*N*: 1,271 peptides) expressing the Hap1/Hap8 variants of ERAP1 (blue), with the B*51:08 ligands from LCL BCH-30 (N: 624 peptides) expressing the Hap10 variant (red). The comparisons involve **(A)** peptide length distribution, **(B)** percent of peptides with Pro2 or Ala2, **(C)** Percent residue frequencies at P1 among peptides with Pro2 (upper panel) or Ala2 (lower panel), **(D)** percent of peptides with P1 residues showing low or intermediate+high susceptibility to ERAP1 trimming among peptides with Pro2 (upper panel) or Ala2 (lower panel), **(E)** theoretical affinity of B*51:01 and B*51:08 ligands for their respective B*51 subtypes, with the medians indicated by bars. The statistical significance of the changes is indicated by the *p*-values, as estimated by the χ^2^ **(A–D)** or Mann–Whitney tests **(E)**. These data were originally published in reference (103).

The following effects were observed in the less active ERAP1 context of B^*^51:08, relative to B^*^51:01 (Figure 4): (1) the percentage of octamers was decreased, and that of nonamers increased, reflecting lower trimming, (2) The Ala2 subpeptidome was increased, at the expense of the Pro2 subpeptidome; the differential distribution of P1 residues in both subpeptidomes was similar in B^*^51:08 and B^*^51:01, but the Ala2 subpeptidome showed a higher percentage of peptides with ERAP1-susceptible residues in B^*^51:08, according to the lower activity of its ERAP1 background, (3) since the Pro2 subpeptidome has higher affinity, this resulted in lower global affinity of the HLA-B^*^51:08 peptidome expressed in the Hap10 context.

### ERAP1 and the HLA-C^*^06:02 peptidome

To my knowledge there are no studies directly addressing the effects of ERAP1 on the C^*^06:02 peptidome. However, based on the similar frequency of ERAP1-susceptible P1 residues (A,C, L, M, Y) in C^*^06:02 compared to HLA-B^*^27 (about 20 and 27%, respectively), it is plausible to predict that the effects of this enzyme on C^*^06:02 will also be significant.

## ERAP2 and the peptidomes of disease-associated MHC-I molecules

Unlike ERAP1, ERAP2 is not expressed in at least 25% of individuals due to frequent polymorphisms that impair protein expression (2, 65). Most ERAP2-positive individuals seem to express a single variant, characterized by the presence of Lys at position 392 (65). Thus, it is the presence or absence of ERAP2 and its expression level, rather than polymorphism altering its enzymatic activity, which associates with disease risk or protection. So far the effects of ERAP2 have been examined on the HLA-B^*^27 and A^*^29:02 peptidomes, since ERAP2 expression is a risk factor for both AS and BSCR.

### ERAP2 and HLA-B^*^27

**Figure 5:**
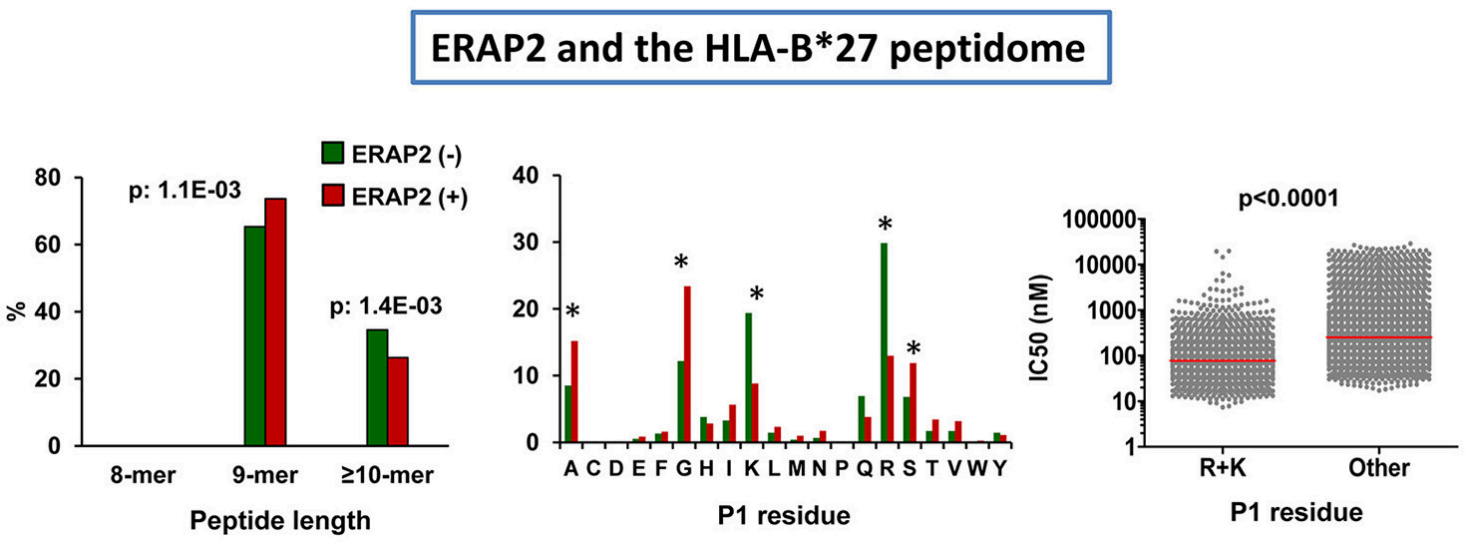
Influence of ERAP2 on the HLA-B*27 peptidome. Comparison of the B*27:05 ligands over-represented (>1.5-fold) in the ERAP2-negative LCL 10151 (N: 764 peptides) expressing the Hap2 variant of ERAP1 (green), with those over-represented in the ERAP2 positive LCL 6370 (N: 817) expressing the same ERAP1 variant (red). The comparisons involve peptide length distribution (left panel), percent frequencies of P1 residues (middle panel), and theoretical affinity of HLA-B*27 ligands (N: 4,945), with N-terminal basic or other residues, with the medians indicated by bars. The statistical significance of the changes is indicated by the *p*-values (left and right panels) or asterisks (middle panel), as estimated by the χ^2^ (two left panels) or Mann–Whitney tests (right panel). The data in the two left panels and those in the right panel were originally published in Martin-Esteban et al. (101, 106), respectively.

The effects of ERAP2 expression on the HLA-B^*^27 peptidome were initially examined on cell lines expressing highly active ERAP1 variants (Hap1 or Hap2), but differing in the expression or not of ERAP2 (106). The presence of this enzyme affected the peptidome at two levels. The first one was an increase in the amounts of 9-mers relative to longer peptides. The second one was a substantial decrease in the amounts of peptides with basic P1 residues (Figure 5), which can be explained by their relatively high frequency among HLA-B^*^27 ligands and their susceptibility to ERAP2 trimming, illustrating the role of this enzyme in epitope destruction. The predominant effect of ERAP2 on HLA-B^*^27 ligands with basic P1 residues strongly argues in favor of a direct effect of the enzyme on trimming, rather than through the allosteric activation of ERAP1, as reported for ERAP1/2 heterodimers *in vitro* (107, 108), since this would presumably induce a more widespread alteration of P1 frequencies. Yet, considering the relatively low efficiency of ERAP2 with long substrates (52), the effects on peptide length are compatible with some improvement of ERAP1 trimming in the presence of ERAP2. Thus, our study did not exclude a functional interaction between both enzymes in the processing of the HLA-B^*^27 peptidome, but suggested that they largely act as separate entities in live cells.

We next addressed the relevance of ERAP2 in distinct ERAP1 contexts (101). When the HLA-B^*^27 peptidomes expressed in a low activity ERAP1 background and presence of ERAP2 (Hap10/ERAP2+) and in a high activity ERAP1 context and absence of ERAP2 (Hap1 or Hap2/ERAP2-) were compared, the changes in peptide length and P1 residue usage reproduced those observed in ERAP2-positive cells differing in the activity of their ERAP1 variants (Hap2 vs. Hap10), but, in addition, the amounts of peptides with basic P1 residues were significantly lower in the presence of ERAP2. This study confirmed the high degree of functional independence of both enzymes *in vivo*, and also revealed an effect of ERAP2 on lowering the affinity of the HLA-B^*^27 peptidome (Figure 5), which was accounted for by the positive contribution of basic P1 residues to HLA-B^*^27 binding (109, 110).

### ERAP2 and HLA-A^*^29:02

**Figure 6:**
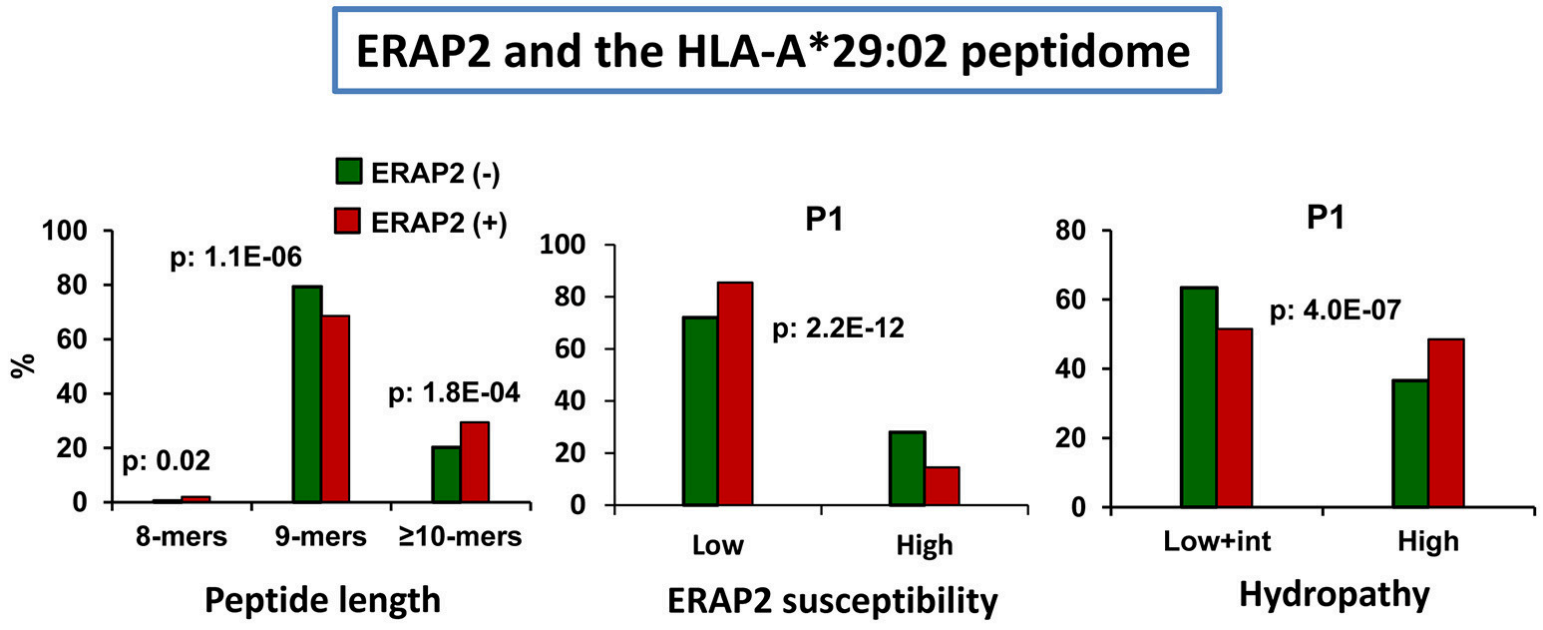
Influence of ERAP2 on the A*29:02 peptidome. Comparison of the A*29:02 ligands over-represented (>1.5-fold) in the mock-transfected ERAP2-negative LCL PF97387 (PG-GFP, N: 839 peptides) expressing the Hap2/Hap3 variants of ERAP1 (green), with those (red) over-represented in the same ERAP2-transfected LCL (PF-ERAP2, N: 990 peptides). The comparisons involve peptide length distribution (left panel), joint frequencies of P1 residues showing low or intermediate+high susceptibility to ERAP2 trimming (middle panel) or showing low+intermediate or high hydropathy (right panel). The statistical significance of the changes is indicated by the *p*-values, as estimated by the χ^2^ test. These data were originally published in reference (93).

The effects of ERAP2 on the A^*^29:02 peptidome have a particular interest due to the association of this enzyme with BSCR (2), a disease exclusively affecting A^*^29:02-positive individuals (3). In a recent study we addressed this issue by comparing the A^*^29:02 peptidomes from two pairs of ERAP1-concordant cell lines differing in the expression or not of ERAP2 and expressing, in all cases, high activity ERAP1 variants (93). Two major effects were observed: (1) the presence of ERAP2 resulted in decreased amounts of 9-mers, relative to longer peptides, and (2) the predominant peptides in the presence of ERAP2 showed lower frequencies of ERAP2-susceptible P1 residues and higher frequencies of hydrophobic ones (Figure 6). These changes did not alter the overall affinity of the peptidome.

The length effects of ERAP2 were opposite to those observed in HLA-B^*^27 and suggested some protection by ERAP2 from ERAP1 trimming, which might be mediated by unproductive binding of long peptides to the former enzyme. That this effect was not observed in HLA-B^*^27 can be explained by the distinct nature of HLA-B^*^27 ligands. These have about two-fold higher frequency of basic P1 residues, which are trimmed by ERAP2 and are ERAP1-resistant, thus diminishing the chances for unproductive binding of non-cognate ligands to ERAP2. In contrast, aliphatic/aromatic P1 residues, which are generally ERAP1-susceptible but ERAP2-resistant are favored and more frequent in A^*^29:02. Moreover, A^*^29:02 ligands are more hydrophobic and might bind better ERAP2 than the more polar HLA-B^*^27 ligands, due to the relatively low electrostatic potential of the substrate binding site of this enzyme (52).

It is interesting to contextualize these effects, along with those of ERAP1 (Figure 3), with the recent genetic findings concerning the combined association of the low activity and decreased expression of the Hap10 variant of ERAP1 and the presence and higher expression level of ERAP2 with BSCR (29). Both low ERAP1 activity and the presence of ERAP2 lead to increased peptide length among A^*^29:02 ligands. Thus, the effects of both risk factors add to each other in this feature. In contrast, the effects on P1 residue frequencies were different: whereas low ERAP1 activity leads to decreased frequency of hydrophobic P1 residues, ERAP2 expression had the opposite effect. These observations might suggest that it is the effect of trimming on peptide length, rather than other features, what relates to BSCR, which in turn might point out to the relevance of particular *uveitogenic* peptide(s), rather than alterations of general features of A^*^29:02, in the pathogenesis of this disease.

Like in HLA-B^*^27, the lower frequency of ERAP2-susceptible, but not of ERAP1-susceptible residues in the presence of ERAP2 argues toward a direct and largely independent effect of this enzyme, relative to ERAP1, on peptide trimming.

The distinct effects of ERAP2 on HLA-B^*^27 and A^*^29:02 indicate that the functional interaction of the enzyme with MHC-I depends on the specific binding preferences of each MHC-I allotype.

### ERAP2 and other disease-associated MHC-I molecules

The effects of ERAP2 on the C^*^06:02 and HLA-B^*^51 peptidomes have not yet been, to my knowledge, experimentally addressed. Based on the P1 residue frequencies of the C^*^06:02 peptidome (Figure 1) it can be inferred that the effects will be smaller than those in HLA-B^*^27 and perhaps closer to those in A^*^29:02, but this issue clearly warrants further research.

Studying the effects of ERAP2 on the HLA-B^*^51 peptidome may be particularly interesting. Unlike other MHC-I-associated diseases, ERAP2 has not been reported to be a risk factor for BD. In addition, the HLA-B^*^51 peptidome (Figure 1) includes very few ligands whose P1 residues are susceptible to ERAP2-trimming, such as basic ones, Gln or Ala (12). Therefore, direct effects of ERAP2 on the HLA-B^*^51 peptidome through over-trimming of B^*^51 ligands are probably very limited. For this reason, this system may be a very suitable one to explore the role of ERAP2 on modulating ERAP1 trimming in live cells, as well as in the generation of MHC-I ligands. The studies are currently ongoing in our laboratory.

## What immunopeptidomics studies teach us about ERAP1 and ERAP2 function

The role of ERAP1 in antigen processing has been widely studied both in mouse and humans. Crystallographic and enzymological studies have greatly contributed to unveiling the mechanism of this enzyme. In addition, multiple *in vitro* studies addressed the role of polymorphic ERAP1 residues, either individually or combined, on peptide trimming and determined how they modulate the enzymatic activity. These issues have been extensively reviewed elsewhere (38, 53, 111–114) Yet, the actual influence of ERAP1 polymorphism in shaping the constitutive peptidomes of MHC-I molecules remained much less explored. This aspect is quite relevant for two reasons. The first one is that ERAP1 is expressed in all individuals but, due to its high polymorphism, the frequency of distinct ERAP1 variants, both within and among populations, is very variable (56). This has likely consequences for the diversity of the antigen handling capabilities among individuals and population groups, a diversity that is amplified by the distinct influence of ERAP1 variants on unrelated MHC-I-bound peptidomes, as discussed above. The second reason is that the discovery that ERAP1 polymorphism is a risk factor for MHC-I associated diseases and is usually in epistasis with the MHC susceptibility allele of each disease, sharpens the focus on the functional interaction between ERAP1 polymorphism and particular disease-associated MHC-I molecules, and therefore on the effects of such polymorphism on MHC-I bound peptidomes. The studies summarized in this review allow us to define the nature of these effects, how they affect the relevant peptidomes, and how they influence the affinity of each peptidome for its cognate MHC molecule. They also allow us to delineate the range of alterations induced by different natural variants of ERAP1, to rank the effects of the individual mutations, which are in turn amplified by linked polymorphisms affecting ERAP1 levels, and to provide a molecular basis for the influence of ERAP1 on distinct MHC-I associated diseases.

The expression pattern of ERAP2 at the population level is much simpler, but not less drastic, than ERAP1. With few exceptions (64) only one enzymatic variant is expressed, and a significant percentage of individuals do not express ERAP2 (65). Due in part to absence of an orthologous enzyme in mice, the role of ERAP2 is less well characterized. Although its structure, substrate specificity and handling features are known from *in vitro* studies (16, 51, 52), the effect of ERAP2 on MHC-I-bound peptidomes in live cells was unknown until its effects on the HLA-B^*^27 and A^*^29:02 peptidomes were analyzed in the studies reviewed above. They revealed that ERAP2 has significant and allotype-dependent effects on both MHC-I molecules, providing a rationale for their association with the diseases for which they are risk factors.

An important issue is the nature of the functional inter-dependence of ERAP1 and ERAP2 in peptide processing. The report that ERAP1/ERAP2 heterodimers existed *in vivo* and account for a fraction of the enzyme pool in the ER (16) suggested that this association could significantly improve the efficiency of their joint hydrolytic activity. Several *in vitro* studies tried to engineer molecular constructs in order to reproduce as closely as possible the heterodimers detected *in vivo*. Such constructs were more efficient than the separate enzymes and their physical interaction apparently induced an activation of ERAP1 (16, 107, 108). Yet, most of the enzyme pool in the ER consists of monomeric molecules and it is unclear to what extent both enzymes physically interact *in vivo*. In addition, the nature of the effects detected on the MHC-I bound peptidomes in live cells can be for the most part explained by separate effects of both enzymes on the peptide pool, and do not seem consistent with a prominent effect mediated by direct activation of ERAP1. The results in live cells do not exclude, and actually support, a functional interdependency of both enzymes in peptide processing. One possible mechanism, suggested by our recent studies on A^*^29:02 (93) and by *in vitro* studies (20) might be based on the unproductive binding of long substrates to ERAP2, impairing or slowing their digestion by ERAP1 on one side, and the capacity of octamers to inhibit ERAP1 trimming of cognate substrates. Digestion of some of these octamers by ERAP2 could counteract their inhibition of ERAP1. The relevance of these effects must be substantiated by direct experimental evidence. However, they may provide a mechanism of functional interaction between both enzymes that obviates the need for a direct activation of ERAP1 by ERAP2, is based on the known enzymatic and peptide handling features of both enzymes, and may be more consistent with the observed alterations of MHC-I-bound peptidomes in live cells.

## What immunopeptidomics studies teach us about the pathogenetic role of MHC-I molecules

MHC-I-associated diseases are polygenic disorders of unknown etiology in which innate and adaptive immune mechanisms, some of which are incompletely understood, are likely to play a role. These diseases involve a variety of inflammatory pathways and affect distinct organs: they are complex pathological conditions in which multiple genetic, immunological, and environmental factors play definite, but poorly characterized roles. The difficulties imposed by this complexity are well illustrated by the fact that, in spite of intensive research for almost 50 years, the pathogenetic role of HLA-B^*^27 in AS remains undefined. Therefore, it is probably naïve to assume that any single-sided methodological or conceptual approach may provide definitive answers to these issues, which can only be obtained by an integrative knowledge of the many factors involved. This fact was suitably stated by Robert and colleagues some years ago in the context of the association of ERAP1 with AS: “*Determining how ERAP1 influences the development of spondyloarthritis may be as complicated as deciphering the role of HLA-B27”* (115). Systems biology approaches, such as that recently employed in preeclampsia (116), may at the end be required for a proper understanding of pathogenetic pathways in these diseases.

Yet, the association of ERAP1 and ERAP2 with distinct MHC-I-associated diseases means that two enzymes that are closely related to MHC-I function, through their processing of MHC-I ligands, play a role in these diseases. This fact strongly suggests that the pathogenetic role of MHC-I molecules is related to their peptide binding features.

Although ERAP1 and ERAP2 are prominent susceptibility factors, the major association is always with the MHC-I molecule. Therefore, these enzymes modulate the strength of, but do not determine, the association of MHC-I with disease. The characterization of their effects on MHC-I-bound peptidomes provides a unique opportunity to define the ways in which the functional interaction between closely related molecules modulates disease susceptibility. Several features of this interaction have so far emerged from immunopeptidomics studies: (1) Both ERAP1 polymorphism and ERAP2 expression affect a seemingly moderate percentage of peptides in a qualitative way, (2) a larger percentage of peptides are either positively or negatively affected in their expression levels by these enzymes, depending mostly on their length and P1 residues, (3) many peptides seem to be unaffected by ERAP1 or ERAP2, either because these enzymes are not involved in their processing or because their generation/destruction balance is the same on distinct enzymatic backgrounds, (4) the alterations in the peptidomes, specially the quantitative ones, significantly re-shape the MHC-I-bound peptidomes depending on the ERAP1/ERAP2 context, (5) these alterations can be large enough to alter, in some cases, the global affinity of the peptidome, (6) the effects are MHC allotype-dependent, since the particular binding specificity of each MHC molecule selects distinct peptide subsets out of those generated in the particular ERAP1/ERAP2 context, and (7) without excluding a functional inter-dependence of ERAP1 and ERAP2, both enzymes seem to act, at least to a large extent, separately *in vivo*.

The alterations in the peptidome may have functional implications at multiple levels: (1) the generation or destruction of specific epitopes in a given ERAP1/2 context has obvious consequences in the antigen presenting capacity of individuals, depending on their particular enzymatic background (117), (2) the quantitative alterations in the peptidome induced by ERAP1/2 polymorphism may, in principle, alter the tolerogenic/autoimmune features of MHC-I molecules since specific T cell activation and immunogenicity depend on epitope density (118, 119), (3) the same alterations and the changes that they induce in the global affinity of the peptidome, may affect NK recognition, which is peptide-dependent and influenced by affinity (120), or otherwise influence NK function (33, 121), and (4) changes in the affinity of the peptidome for the MHC-I molecule may potentially affect its folding kinetics and its stability at the cell surface. In HLA-B27, both folding and surface dissociation leading to generation of non-canonical forms, such as heavy chain homodimers, are thought to influence the pathogenetic potential of this molecule (122–124). Dissecting the role of each of these factors in the different diseases must await further research.

The association of ERAP1/2 with distinct MHC-I-dependent inflammatory diseases initially suggested that similar mechanism(s) might underlie the joint association of ERAP1/2 and MHC-I to different diseases (12). However, the distinct effects of ERAP1/ERAP2 on different MHC-I molecules revealed by recent immunopeptidomics studies may be more compatible with the alternative possibility that the MHC allotype-specific effects of these enzymes actually lead to distinct pathogenetic mechanisms of ERAP/MHC interaction in different diseases. This is also supported by the distinct association patterns of ERAP1 (Table 1). Similar ideas have been proposed by McGonagle and colleagues from a consideration of the pathology/immunopathology of some of these diseases (13).

Finally, the notorious difference in the genetic association of ERAP1 and ERAP2 with disease, namely the epistasis of ERAP1 and the susceptibility MHC-I allele in AS, psoriasis and BD, the non epistatic association of ERAP2 in the two former diseases, and, as far as it is known, the lack of association of ERAP2 with BD, may be better explained by the separate activities of both enzymes on the MHC-I peptidomes, than by assuming a strong influence of ERAP2 on ERAP1 activity.

## Author contributions

The author confirms being the sole contributor of this work and has approved it for publication.

### Conflict of interest statement

The author declares that the research was conducted in the absence of any commercial or financial relationships that could be construed as a potential conflict of interest.
